# Supplementary material for: The interactome of intact mitochondria by cross-linking mass spectrometry provides evidence for coexisting respiratory supercomplexes
Source: Mol Cell Proteomics. 2017 Dec 8;17(2):216–32. doi: 10.1074/mcp.RA117.000470 (PMC5795388; doi:10.1074/mcp.RA117.000470)
Supplement: Supplemental Data [file supp_RA117.000470_133922_0_supp_23978_fzffwf.zip › spectra_annotation/mito_DR_spectra_annotation/360-1-2-1-2-1.pdf]

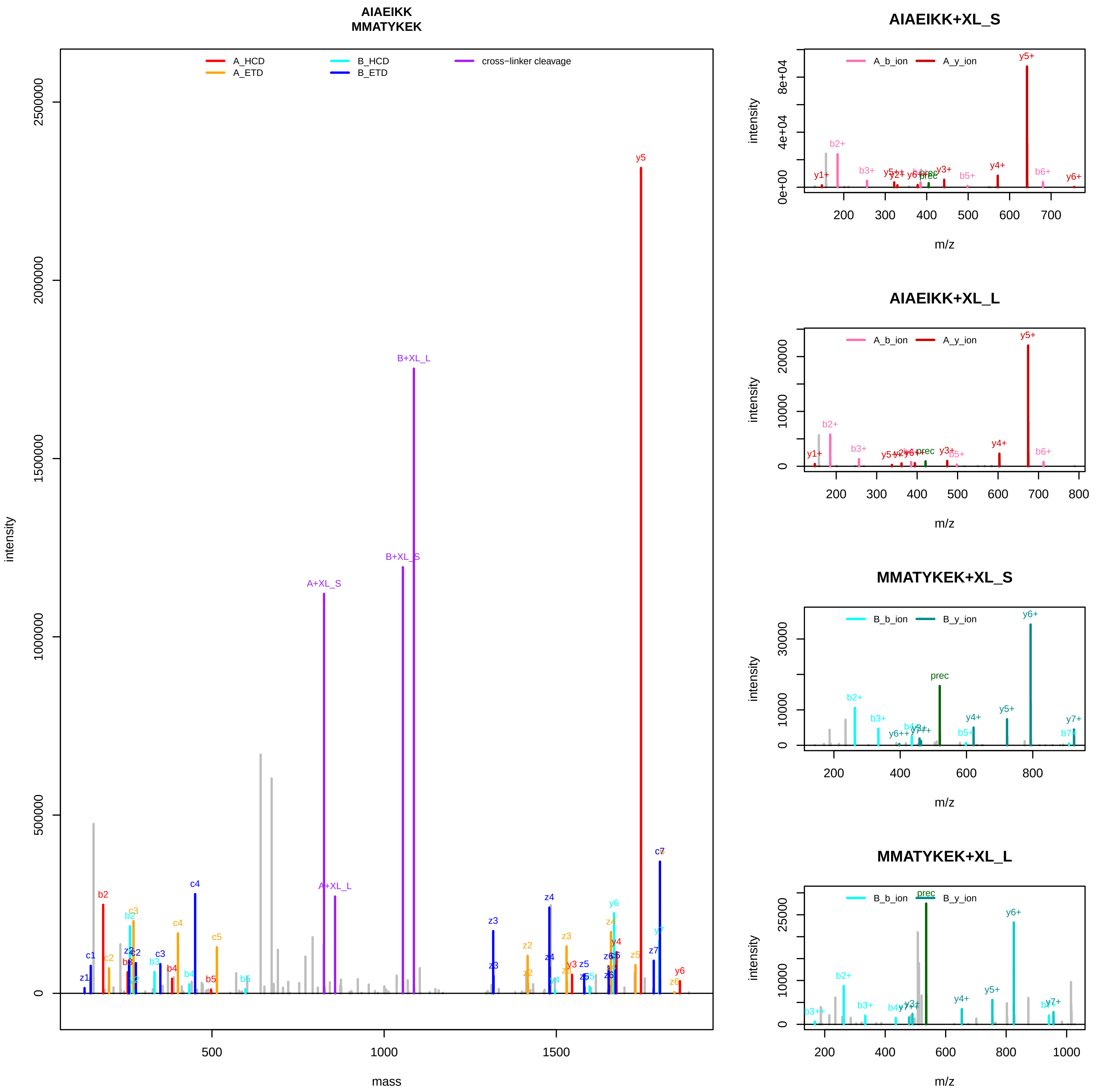

AIAEIKK
MMATYKEK
A_HCD
A_ETD
B_HCD
B_ETD
cross−linker cleavage
intensity
mass
0
500000
1000000
1500000
2000000
2500000
500
1000
1500
y5
B+XL_L
B+XL_S
A+XL_S
A+XL_L
c7
z7
y7
y6
z6
z5
z4
z3
c5
c4
c3
c2
c1
z1
z2
b2
b3
b4
b5
y3
y4
z4
AIAEIKK+XL_S
A_b_ion
A_y_ion
y5+
b2+
b3+
y1+
y4+
y3+
b5+
b6+
y6+
prec
m/z
0e+00
4e+04
8e+04
AIAEIKK+XL_L
0
10000
20000
MMATYKEK+XL_S
B_b_ion
B_y_ion
y6+
prec
b2+
b3+
y4+
y5+
y7+
b5+
b7+
30000
MMATYKEK+XL_L
y4+
y5+
b3++
b4+
25000
